# Proposal of a post-prostatectomy clinical target volume based on pre-operative MRI: volumetric and dosimetric comparison to the RTOG guidelines

**DOI:** 10.1186/s13014-014-0303-6

**Published:** 2014-12-23

**Authors:** Jennifer Croke, Jillian Maclean, Balazs Nyiri, Yan Li, Kyle Malone, Leonard Avruch, Cathleen Kayser, Shawn Malone

**Affiliations:** The Ottawa Hospital Cancer Centre, K1H 8 L6 Ottawa, Ontario Canada

**Keywords:** Prostate cancer, Post-prostatectomy, Adjuvant, Salvage radiotherapy, MRI, Consensus guidelines, CTV

## Abstract

**Background:**

Recurrence rates following radiotherapy for prostate cancer in the post-operative adjuvant or salvage setting remain substantial. Previous work from our institution demonstrated that published prostate bed CTV guidelines frequently do not cover the pre-operative MRI defined prostate. Inadequate target delineation may contribute to the high recurrence rates, but increasing target volumes may increase dose to organs at risk.

**Methods:**

We propose guidelines for delineating post-prostatectomy target volumes based upon an individual’s co-registered pre-operative MRI. MRI-based CTVs and PTVs were compared to those created using the RTOG guidelines in 30 patients. Contours were analysed in terms of absolute volume, intersection volume (Jaccard Index) and the ability to meet the RADICALS and QUANTEC rectal and bladder constraints (tomotherapy IMRT plans with PTV coverage of V98% ≥98%).

**Results:**

CTV MRI was a mean of 18.6% larger than CTV RTOG: CTV MRI mean 138 cc (range 72.3 - 222.2 cc), CTV RTOG mean 116.3 cc (range 62.1 - 176.6 cc), (p < 0.0001). The difference in mean PTV was only 4.6%: PTV MRI mean 386.9 cc (range 254.4 – 551.2), PTV RTOG mean 370 cc (range 232.3 - 501.6) (p = 0.05). The mean Jaccard Index representing intersection volume between CTVs was 0.72 and 0.84 for PTVs. Both criteria had a similar ability to meet rectal and bladder constraints. Rectal DVH: 77% of CTV RTOG cases passed all RADICALS criteria and 37% all QUANTEC criteria; versus 73% and 40% for CTV MRI (p = 1.0 for both). Bladder DVH; 47% of CTV RTOG cases passed all RADICALS criteria and 67% all QUANTEC criteria, versus 57% and 60% for CTV MRI (p = 0.61for RADICALS, p = 0.79 for QUANTEC). CTV MRI spares more of the lower anterior bladder wall than CTV RTOG but increases coverage of the superior lateral bladder walls.

**Conclusion:**

CTV contours based upon the patient’s co-registered pre-operative MRI in the post-prostatectomy setting may improve coverage of the individual’s prostate bed without substantially increasing the PTV size or dose to bladder/ rectum compared to RTOG CTV guidelines. Further evaluation of whether the use of pre-operative MRI improves local control rates is warranted.

## Introduction

15-25% of all patients who undergo radical prostatectomy relapse and, amongst those with high risk features (positive margins, T3 disease), the rate of biochemical recurrence (BCR) within 5 years is 45-75% [1-3]. Randomised studies consistently report improved rates of local control and biochemical progression free survival in high risk prostate cancer patients following adjuvant radiotherapy [4-6], with one study reporting improved metastasis-free and overall survival [5]. Whether early salvage radiotherapy at the first indication of BCR yields outcomes equivalent to adjuvant treatment remains the subject of further randomised studies.

However, despite adjuvant or salvage post-operative radiotherapy, a large proportion of patients with high risk disease recur. Mature data from patients who received adjuvant radiotherapy within the above studies report BCR rates of 40-50% at 10 years. SWOG 8794 indicated that the pattern of treatment failure in high-risk patients is predominantly local even with adjuvant radiotherapy: 20% of the surgery only arm experienced local relapse versus 7% of patients undergoing adjuvant radiotherapy (12% versus 4% developed distant relapse) [7]. EORTC 22911 reported similar findings with long-term local relapse identified in 7% of patients in the adjuvant radiotherapy arm (a further 7.2% experienced metastatic disease) [4]. Local failure post-radiotherapy results from either inherent tumour radio-resistance/suboptimal dose or inadequate target delineation. Whilst it can be argued that the radiotherapy delivered in these studies was suboptimal by modern standards, the increasing use of IMRT in an attempt to spare organs at risk could paradoxically increase the local recurrence risk if the target is not appropriately contoured.

It is essential that the post-operative clinical target volume (CTV) includes the entire prostate bed and planes of surgical dissection as these are the areas at greatest risk of possessing microscopic disease [8-10]. Four published consensus guidelines have variously defined the post-operative CTV in prostate cancer (European Organization for Research and Treatment of Cancer [EORTC], Faculty of Radiation Oncology Genito-Urinary Group [FROGG], Princess Margaret Hospital [PMH], and Radiation Therapy Oncology Group [RTOG]) [11-14]. The CTV borders in each of these guidelines are based upon anatomical landmarks and important differences exist between guidelines. Only the EORTC guidelines specifically take account of the pathology report, recommending a 5 mm expansion in the direction of a positive margin, although the CTV FROGG makes reference to surgical clips. Previous work from our institution showed that none of the four guidelines adequately covered the prostate bed and/or gross tumour based on preoperative MRI in a non-select group of 20 patients [15]. On average, 38% of the prostate volume and 41% of gross tumour volume on pre-operative MRI were not included in the CTV. The CTV RTOG and CTV PMH provided the best coverage with the CTV-EORTC being the least adequate. This suggests that improved target delineation could potentially improve outcomes.

We therefore propose an alternative CTV for post-operative radiotherapy to the prostate bed that incorporates information from each patient’s pre-operative MRI (CTV MRI) and have evaluated this in comparison to the RTOG guidelines in terms of target volume and doses to the rectum and bladder.

## Methods

### Patient cohort

The clinical study was approved by the institution’s Research Ethics Board. A list of prostate cancer patients who underwent radical prostatectomy (RP) and adjuvant or salvage radiotherapy from May 2007 to January 2011 at our institution was compiled and the first 30 patients (chosen alphabetically) with pre-operative staging pelvic MRI comprised the study group. The decision for pre-operative MRI staging had been at the discretion of the treating Urologist. Patients were excluded if they had an endo-rectal coil placed during the preoperative MRI as the probe can result in compression and displacement of the prostate.

### Planning CT and MRI parameters

All patients had undergone radiotherapy treatment planning CT scans in a supine position with a rubber leg cushion immobilization device and full bladder/empty rectum (achieved via a fleet enema prior to CT Simulation). The planning CT scan extended from 5 cm above the L5-S1 vertebral body to 5 cm below the ischial tuberosity using 3 mm axial slices. Planning CT scans for the 30 patients were imported into a CMS Focal planning station for contouring.

Pre-operative MRIs were performed on either a Siemens Trio Tim (3 T), Symphony Tim (1.5 T) or Symphony (1.5 T) scanner. 3-5 mm T2-weighted turbo spin echo and 4-5 mm T1-weighted fat saturation fast low angle shot pre- and post-gadolinium (at 1 and 2 min) scans were available in the axial, coronal and sagittal planes. Axial T1 post-gadolinium and T2 images were co-registered to the planning CT scan using the “auto fusion” function and adjusted manually as required to provide optimal fusion in all three planes using pelvic bone landmarks.

### Contouring

Two CTVs were contoured for each patient: the first CTV followed the RTOG Consensus guideline (CTV RTOG) and the second was based on the pre-operative MRI (CTV MRI). Two radiation oncologists (JC and SM) jointly contoured all of the targets and the MRI contours were reviewed by an expert MRI radiologist (LA) to ensure accurate delineation. Furthermore, for quality assurance purposes, the CTV RTOG guideline was reviewed in detail at the initiation of this study by the 7 Radiation Oncologists specialising in genitourinary (GU) malignancy at our institution. For each patient the following landmarks were contoured on the treatment planning CT Scan: rectum, bladder, vesicourethral anastomosis, penile bulb, and bladder neck. The CTV RTOG was contoured for each patient according to the protocol summarised in Table 1 using the planning CT scan only. Where the RTOG guidelines specified a range of possible anatomical borders, the largest was chosen in each case to provide the most generous CTV.

**Table 1:** **RTOG Consensus CTV Guidelines**

**Location**	**Anatomical border**	
	**Below superior edge of pubic symphysis**	**Above superior edge of pubic symphysis**
**Anterior**	Posterior edge of pubic bone	Posterior 1-2 cm of bladder wall
**Posterior**	Anterior rectal wall (may need to be concave around lateral aspects)	Mesorectal fascia
**Lateral**	Levator ani muscles, obturator internus	Sacrorectogenitopubic fascia (if concern about extraprostatic disease at base may extend to obturator internus)
**Inferior**	8-12 mm below vesicourethral anastomosis (may include more if concern for apical margin. Can extend to slice above penile bulb if vesicourethral anastomosis not well visualized)	N/A
**Superior**	N/A	Level of cut end of vas deferens or 3-4 cm above top of symphysis (Vas may retract postoperatively; include seminal vesicle remnants if pathologically involved)

### CTV MRI

The prostate, seminal vesicles and any gross visible tumour were contoured using the co-registered MRI. Gross tumour was identified as a hypointense lesion on T2 MRI and/or enhancing tumour on T1 post -gadolinium MRI.

The CTV MRI was created as follows:

1. The prostate and any gross visible tumour extending outwith the prostate was expanded by 5 mm to account for potential extra-prostatic extension
2. This volume was tailored to respect anatomic barriers including the fascia of the pelvic muscles, the periosteum of the pubic symphysis and Denovillier’s fascia along the anterior rectum.
3. In cases where there was a geographic shift of the rectum between the pre-operative MRI and the post-operative CT simulation, CTV MRI was tailored to exclude the anterior rectal wall. In these cases CTV MRI was extended laterally to include the peri-rectal fat space that could harbour microscopic disease (including the pre-operative MRI defined prostate plus 5 mm).
4. To include the planes of surgical dissection the CTV MRI was extended to include all surgical clips and to the medial fascia of the levator ani/obturator internus muscle.
5. CTV MRI was trimmed superiorly where there was a prominent median lobe (benign prostatic hypertrophy) projecting into the bladder. In these cases the inferior bladder wall lies adjacent to the prostate and there is no adjacent peri-prostatic fat. During radical prostatectomy the bladder neck is pulled down inferiorly and attached to the urethra. In cases where there is pelvic fat adjacent to the base of the prostate and or gross tumour, CTV MRI was not tailored. For these cases, the fat space remains attached to the pelvic side wall and pubic symphysis and is at risk for harbouring microscopic residual disease.
6. Seminal vesicle coverage was done in accordance with the CTV RTOG guideline.

### PTV

PTV RTOG and PTV MRI were created using a 1 cm isotropic expansion of the respective CTVs.

### Treatment planning

Two tomotherapy IMRT plans were created for all 30 cases (PTV RTOG and PTV MRI). A dose of 6600 cGy in 33 fractions was used to cover the PTV, as per the RADICALS protocol [16]. Inverse planning was performed using Tomotherapy Planning Software (TPS) Version 4.0. Plans were optimized to minimize both rectal and bladder doses and to provide PTV coverage of V98% ≥100%. A beam width of 2.5 cm and a pitch of 0.287 were used for each plan.

### Analysis

The mean and range volumes for the CTVs and PTVs were calculated and the statistical significance of the difference in absolute volumes assessed using a paired t-test. The Jaccard Index was calculated for CTVs and PTVs in each case to provide a measure of the similarity between volumes. This is derived by dividing the intersection volume of CTV RTOG and CTV MRI (or PTV) by the union volume of both CTVs (or PTVs). A Jaccard Index of 1 equates to 100% overlap and 0 means no overlap:

$$ \mathrm{Jaccard}\ \mathrm{I}\mathrm{ndex} = \frac{\mathrm{CTV}\ \mathrm{R}\mathrm{T}\mathrm{OG}\ \cap\ \mathrm{C}\mathrm{T}\mathrm{V}\ \mathrm{M}\mathrm{R}\mathrm{I}}{\mathrm{CTV}\ \mathrm{R}\mathrm{T}\mathrm{OG}\cup \mathrm{C}\mathrm{T}\mathrm{V}\ \mathrm{M}\mathrm{R}\mathrm{I}} $$

Dose volume histograms (DVHs) were evaluated for ability to meet the desired target coverage and OAR (rectum and bladder) dose constraints set forth by RADICALS [16] and QUANTEC [17] guidelines (Table 2) which were recorded as pass/fail. The statistical significance of differences in the ability to meet dose constraints of each PTV was compared using the Fisher’s exact test.

**Table 2:** **Relevant DVH Constraints for Rectum and Bladder in RADICALS and QUANTEC**

**Constraint**	**Rectum**		**Bladder**	
**(Gy)**	**RDICALS**	**QUANTEC**	**RADICALS**	**QUANTEC**
30	<80%	-	-	-
40	<70%	-	-	-
50	<60%	<50%	<80%	-
60	<50%	<35%	<50%	-
65	-	<25%	-	<50%
66	<30%	-	-	-
70	-	<20%	-	<35%

## Results

### Target volume

**Figure 1:**
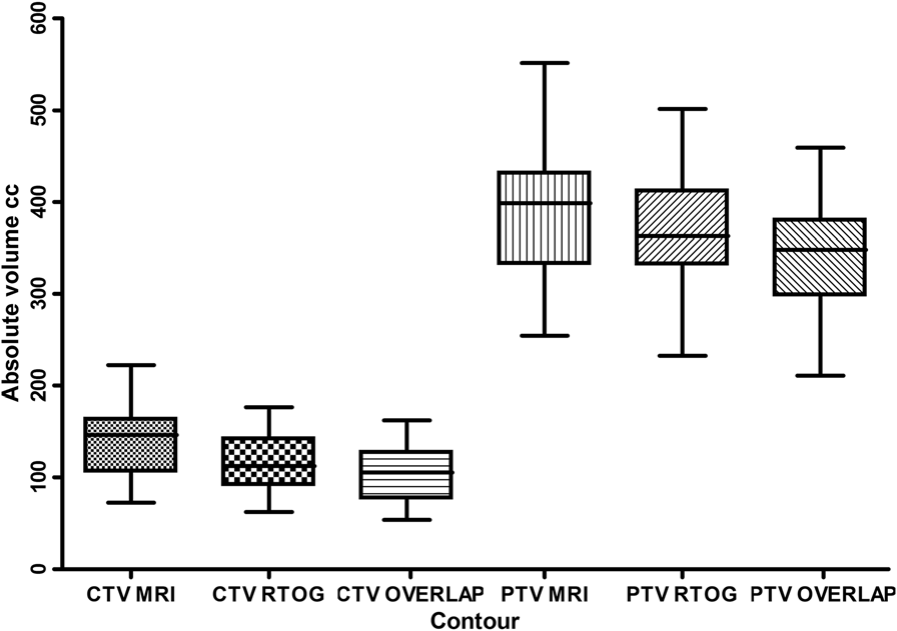
**Box and whiskers plot for absolute and overlap CTV/ PTVs.** The whiskers are located at the maximum and minimum values; the box shows the 25-75^th^ centiles with the median marked.

**Figure 2:**
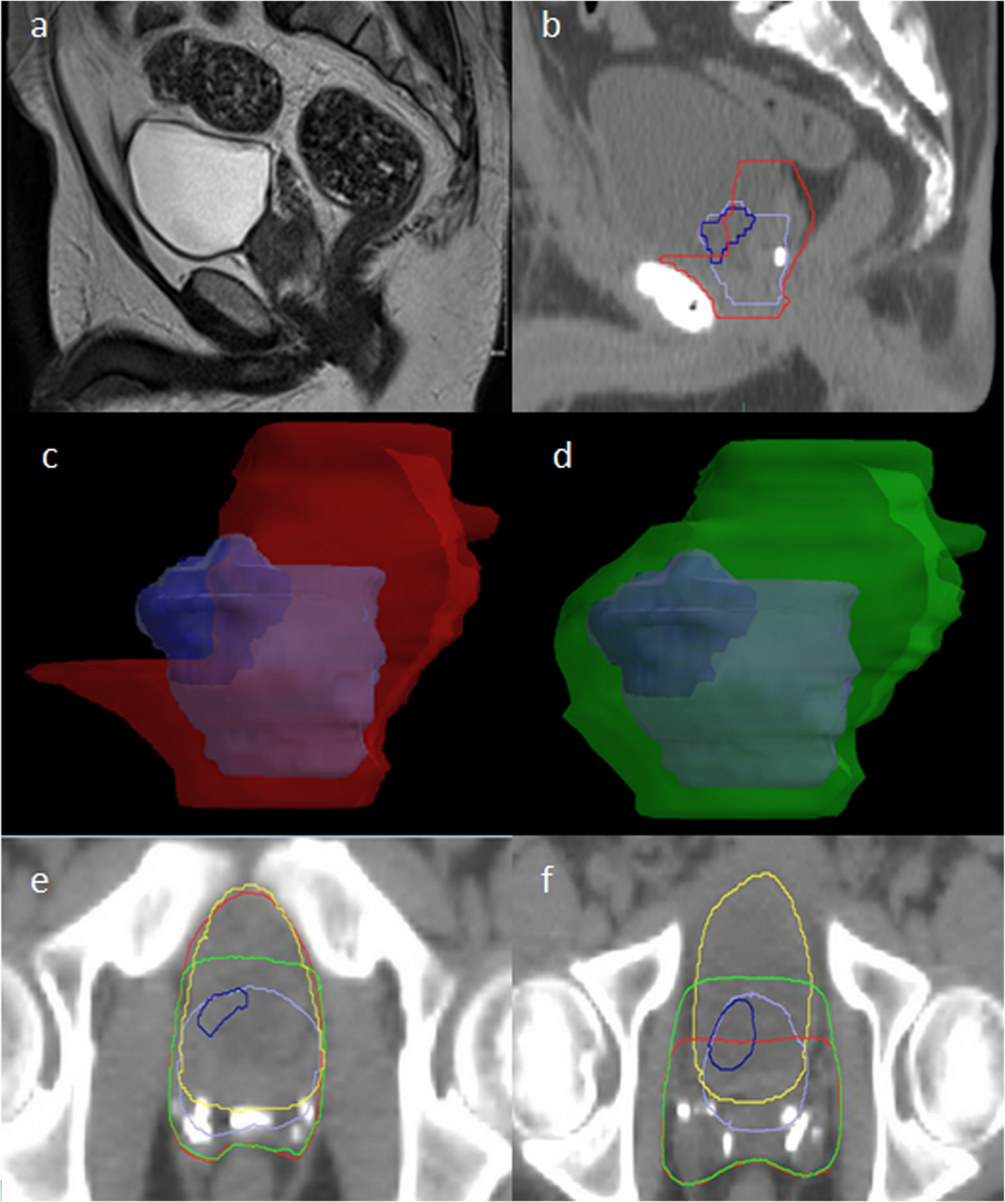
**Typical case depicting the differences in shape and coverage between CTV RTOG and CTV MRI.** Key - Purple: pre-op prostate, Blue: pre-op visible tumour, Red: CTV RTOG, Green: CTV MRI, Yellow: bladder. **(a)** pre-operative T2 MRI **(b)** planning CT scan (post-operative); the bladder neck has been pulled down. **(c)** CTV RTOG does not completely cover the region of the pre-operative prostate/ tumour. Although the bladder will now largely fill the location of the original prostate, the lateral soft tissue lateral remains at risk of microscopic disease. **(d)** CTV MRI extends 5 mm around the original prostate to cover the soft tissue that was adjacent to the prostate **(e)** Inferiorly the CTV RTOG treats more of the anterior bladder wall than CTV MRI where coverage may not be required in view of the original prostate location. **(f)** Superiorly the CTV MRI increases dose to the lateral bladder walls because it covers the soft tissue beside the bladder that remains at risk of microscopic disease in view of the original prostate/tumour location. CTV RTOG misses these areas.

The CTV MRI was a mean of 18.6% larger than CTV RTOG (larger in 26 cases): CTV MRI mean 138 cc (range 72.3 - 222.2 cc), CTV RTOG mean 116.3 cc (range 62.1 - 176.6 cc), (p < 0.0001). The addition of the isotropic PTV margins reduced this difference to only 4.6% (PTV MRI larger in 21 cases); PTV MRI mean 386.9 cc (range 254.4 – 551.2), PTV RTOG mean 370 cc (range 232.3 - 501.6), (p = 0.05). The mean Jaccard Index was 0.72 for CTV (standard deviation 0.09) and 0.84 for PTV (SD 0.06). Graphical depiction of absolute and overlap volumes is shown in Figure 1. Figure 2 shows a typical example of differences in the volumes for one case.

The desired PTV coverage of V98% of 98% was met in all cases for both PTV RTOG and PTV MRI.

### Organs at risk

Table 3 details the adherence to each OAR constraint for plans created to cover the PTVs created using CTV RTOG and CTV MRI. Both failed to meet the OAR dose constraints proposed by RADICALS and QUANTEC in a considerable number of cases and there were no significant differences between either contouring criteria. For the rectal DVH: 77% of CTV RTOG cases passed all the RADICALS criteria and 37% all the QUANTEC criteria; versus 73% and 40% for CTV MRI (p = 1.0 for both). For the bladder DVH; 47% of CTV RTOG cases passed all the RADICALS criteria and 67% all the QUANTEC criteria versus 57% and 60% for CTV MRI (p = 0.61for RADICALS and p = 0.79 for QUANTEC). Plans created using CTV MRI generally spare more of the inferior anterior bladder wall compared to CTV RTOG; however, CTV MRI results in greater treatment of the lateral bladder wall (Figure 2). Both CTVs were similarly better able to meet the V50 proposed by RADICALS compared to V60 (p = 1.0).

**Table 3:** **Percentage of cases where the plan created for PTV RTOG and PTV MRI passed the criteria**

	**% Passing Criteria (n)**							
	**Rectum**				**Bladder**			
	**RADICALS**		**QUANTEC**		**RADICALS**		**QUANTEC**	
**Constraint**	**RTOG**	**MRI**	**RTOG**	**MRI**	**RTOG**	**MRI**	**RTOG**	**MRI**
V30	83 (25/30)	77 (23/30)	-	-	-	-	-	-
V40	93 (28/30)	97 (29/30)	-	-	-	-	-	-
V50	93 (28/30)	83 (25/30)	40 (12/30)	60 (18/30)	73 (22/30)	70 (21/30)	-	-
V60	93 (28/30)	90 (27/30)	37 (11/30)	40 (12/30)	47 (14/30)	57 (17/30)	-	-
V65	-	-	-	-	-	-	67 (20/30)	60 (18/30)
V66	77 (23/30)	73 (22/30)	-	-	-	-	-	-

## Discussion

When post-operative radiotherapy is undertaken the delineation of an appropriate CTV is integral to treatment success regardless of whether this is delivered in the adjuvant or salvage setting. In other cancer sites, radiation oncologists generally aim to cover the entire surgical bed and use information from surgical and pathology reports as well as pre and post-operative imaging to define the CTV. However, the four published post-operative prostate radiation CTV guidelines are largely based on anatomic boundaries rather than the individual’s surgical bed and significant volumetric differences exist between guidelines [15].

The rationale for post-operative radiotherapy in high risk patients is that the majority of initial post-prostatectomy relapses appear to be local [7]. However, randomised studies of post-prostatectomy radiotherapy still report high relapse rates following radiotherapy. Ten year follow-up in SWOG 8794 reported BCR in 42% of post-prostatectomy patients with pre-treatment PSAs ≤0.2 who had undergone adjuvant radiotherapy (7% confirmed local disease, 4% confirmed metastatic disease) [5]. Similarly, after the same median follow-up in EORTC 22911 39.4% of patients had BCR, clinical progression or had died and PFS rates in the ARO 96-02 study were 56% (BCR, local or metastatic disease) [4,6]. This suggests inadequacies in radiotherapy delivery or dose. It should be emphasised that of these adjuvant radiotherapy studies, only ARO 96-02 used CT-based contouring/ planning. Therefore, adherence to any of the four current CT-based CTV consensus guidelines could reasonably be expected to result in better target coverage. On the other hand, our previous work suggested that the consensus guidelines did not adequately cover the prostate bed and the move towards IMRT as the standard method of delivering post-prostatectomy radiotherapy therefore has the potential to result in more treatment failures [15].

Malignant cells located in the extra-prostatic fat space (at sites of extra-prostatic extension and positive margins) are unlikely to be pulled down with the bladder during prostatectomy and the current guidelines for CTV delineation may not adequately cover this region as it is dependent on individual anatomy. We have therefore proposed an individualised post-operative CTV based on the patient’s pre-operative MRI. The RTOG guidelines were chosen as the comparator as they best covered the prostate bed in our previous study. The paper detailing the RTOG guidelines acknowledges that pre-operative imaging can help establish the superior extent of the CTV, but makes no specific recommendations regarding this [14].

Extra-prostatic extension typically extends within 5 mm of the prostate [18,19], hence our 5 mm CTV expansion beyond the MRI defined prostate. Coverage of seminal vesicles was done in accordance to the RTOG guideline. As per the ICRU definition of CTV, we restricted our CTV definition to anatomic boundaries. Furthermore, coverage of the prostate base was modified in cases where there was a prominent median lobe suggestive of benign prostatic hypertrophy. We included the fat space lateral to the prostate up to the levator ani as these tissues are not pulled down with the bladder during prostatectomy and therefore are at risk for harbouring microscopic disease. Our previous research indicated that the predominant site of geographic miss using the four consensus guidelines was the base region of the prostate [15]. Wang et al., recently evaluated regions of local recurrence after RP in relation to whether these would have been covered using the RTOG guidelines [20]. They reported that RTOG CTV contours did not appear adequate posterolaterally near the rectum/mesorectal fascia and inferiorly at the posterior urogenital diaphragm. Use of the CTV MRI should improve coverage of such regions.

CTV MRI was larger in terms of absolute volume than CTV RTOG, but this difference was <5% when isometric PTV margins were applied. However, there was a more significant difference between contours in volumetric shape (a mean of 16% of PTV was outside of the overlap volume). CTV RTOG extends to the top of the pubic symphysis anteriorly, while CTV MRI is tailored in this region to follow the preoperative prostate and sites of potential extra-prostate spread (prostate plus 5 mm). As a result of this shape difference CTV MRI is able to spare the inferior bladder wall anteriorly as compared to CTV RTOG. Adequate coverage of the pre-operative prostate base within CTV MRI increased dose to the superior portion of the lateral bladder walls compared to CTV RTOG, but IMRT plans created for both PTVs met the dose constraints proposed by RADICALS and QUANTEC in a similar percentage of cases. This is reassuring in that it indicates that the use of CTV MRI should not result in greater normal tissue toxicity than CTV RTOG. However, both CTVs failed to meet all the rectal and bladder constraints in a large percentage of cases. It should be noted of course that a large percentage of the bladder in the treatment field is not bladder wall and treatment of the urine-filled volume is unlikely to cause toxicity. There is currently a paucity of clinical data validating current DVH guides in the post-operative setting. Therefore, as the CTV MRI clearly defines regions at high risk of relapse for the individual, whether it is appropriate to compromise CTV coverage to meet all of the OAR dose constraints needs careful consideration. Information from ongoing randomised clinical trials will help researchers to define optimal DVHs for OARs in this patient population.

There are various limitations in this study in relation to the use of MRI. MRI and planning CT were automatically co-registered in relation to bony contours and manually adjusted as required, as described in reports of CT/MRI coregistration in the primary radiotherapy setting [21,22]. There are inherent limitations to such co-registrations in the pelvis due to variable bowel and bladder filling. Such uncertainties are likely increased in the post-prostatectomy setting as periprostatic tissue may move relative to the bones and we noted changes in rectal wall position in many patients. However, the CTV MRI was tailored accordingly and daily image guidance should be used during radiotherapy to offset interfraction organ position changes as much as possible. Secondly, there remains debate regarding the optimal MRI sequences in prostate cancer and there can be significant interobserver variability in image interpretation/contouring. As such we chose to co-register simple and widely available T1 post-contrast and T2 series. These are familiar to most prostate radiation oncologists and our MRI contours were reviewed by an expert MRI pelvis radiologist. As we were largely interested in the superficial anatomy of the prostate, it is unlikely that more advanced MRI sequences or 3 Tesla imaging would have significantly improved prostate bed coverage and less familiar sequences could have added to interpretation uncertainties. Furthermore, interobserver variability is not unique to MRI - the delineation of the post-prostatectomy target volume using the EORTC consensus guidelines (without MRI) showed only moderate observer agreement [23]. It would be interesting to evaluate contouring consistency using CTV MRI against any of the consensus guidelines in a future study. Endorectal coils were not used in our patients as they can deform the prostate and are thus avoided when co-registration for radiotherapy is anticipated. It is important that centres consider this when formulating imaging protocols as several studies indicate that endorectal coils improve diagnostic image quality and their use is recommended in the ESUR prostate cancer staging protocol [24].

Finally, a fundamental issue that could hamper the widespread adoption of CTV MRI is the considerable disparity that remains in many regions regarding routine MRI use, despite the fact that MRI is increasingly recommended in prostate cancer guidelines. MRI is more accurate than DRE at defining prostate T stage, better correlates with post-radiation outcomes and provides important nodal evaluation in higher risk patients [24-27]. Furthermore, MRI-based radiotherapy target volumes are smaller than CT-based targets with less inter/intra-observer variability [21,28] and MRI may also improve surgical planning, particularly in terms of whether to spare or resect the neurovascular bundles [29,30]. As such, the 2012 European Society of Urogenital Radiology (ESUR) guidelines conclude that multiparametric MRI is an integral part of prostate cancer diagnosis and management for all risk categories of patients, including those considering active surveillance [24]. Additionally, the UK National Institute of Health and Care Excellence (NICE) guidelines, which consider clinical benefit and cost effectiveness, recommend a multiparametric MRI scan in men with histologically proven prostate cancer when knowledge of the T or N stage could affect management [31] (effectively all candidates for radical treatment/active surveillance). However, the 2013 American College of Radiology ACR Appropriateness Criteria® support routine MRI only in patients with intermediate to high risk prostate cancer and for some low-risk cases prior to active surveillance [32]. Regardless, this should have limited impact on the potential for CTV MRI use as very few patients deemed to be low risk by other parameters are likely to require post-operative radiotherapy.

## Conclusion

This study proposes a post-prostatectomy CTV tailored to the individual by using the co-registered pre-operative MRI, which appears to better cover each individual’s prostate bed without substantially increasing the PTV size. Volumetric shape differences between CTV RTOG and CTV MRI result in different locations of the bladder wall receiving high dose radiation. However, this did not impact upon the ability of CTV MRI to meet currently used OAR dose constraints. This suggests that the use of CTV MRI would be a safe approach to post-prostatectomy radiotherapy targeting. Whether the use of CTV MRI would translate into improved patient outcomes remains a hypothesis that would have to be tested within a prospective clinical study.

## References

[1] Moul JW (2000). Prostate specific antigen only progression of prostate cancer. J Urol.

[2] Stephenson AJ, Scardino PT, Eastham JA, Bianco FJ, Dotan ZA, Fearn PA, Kattan MW (2006). Preoperative nomogram predicting the 10-year probability of prostate cancer recurrence after radical prostatectomy. J Natl Cancer Inst.

[3] Zietman AL, Coen JJ, Shipley WU, Willett CG, Efird JT (1994). Radical radiation therapy in the management of prostatic adenocarcinoma: the initial prostate specific antigen value as a predictor of treatment outcome. J Urol.

[4] Bolla M, van Poppel H, Tombal B, Vekemans K, Da Pozzo L, de Reijke TM, Verbaeys A, Bosset JF, van Velthoven R, Colombel M, van de Beek C, Verhagen P, van den Bergh A, Sternberg C, Gasser T, van Tienhoven G, Scalliet P, Haustermans K, Collette L, European Organisation for R, Treatment of Cancer RO, Genito-Urinary G (2012). Postoperative radiotherapy after radical prostatectomy for high-risk prostate cancer: long-term results of a randomised controlled trial (EORTC trial 22911). Lancet.

[5] Thompson IM, Tangen CM, Paradelo J, Lucia MS, Miller G, Troyer D, Messing E, Forman J, Chin J, Swanson G, Canby-Hagino E, Crawford ED (2009). Adjuvant radiotherapy for pathological T3N0M0 prostate cancer significantly reduces risk of metastases and improves survival: long-term followup of a randomized clinical trial. J Urol.

[6] Wiegel T, Bartkowiak D, Bottke D, Bronner C, Steiner U, Siegmann A, Golz R, Storkel S, Willich N, Semjonow A, Stockle M, Rube C, Rebmann U, Kalble T, Feldmann HJ, Wirth M, Hofmann R, Engenhart-Cabillic R, Hinke A, Hinkelbein W, Miller K. Adjuvant Radiotherapy Versus Wait-and-See After Radical Prostatectomy: 10-year Follow-up of the ARO 96-02/AUO AP 09/95 Trial. European urology. 2014. 10.1016/j.eururo.2014.03.011 24680359

[7] Swanson GP, Hussey MA, Tangen CM, Chin J, Messing E, Canby-Hagino E, Forman JD, Thompson IM, Crawford ED (2007). Swog. Predominant treatment failure in postprostatectomy patients is local: analysis of patterns of treatment failure in SWOG 8794. J Clin Oncol Off J Am Soc Clin Oncol.

[8] Sella T, Schwartz LH, Swindle PW, Onyebuchi CN, Scardino PT, Scher HI, Hricak H (2004). Suspected local recurrence after radical prostatectomy: endorectal coil MR imaging. Radiology.

[9] Kassabian VS, Bottles K, Weaver R, Williams RD, Paulson DF, Scardino PT (1993). Possible mechanism for seeding of tumor during radical prostatectomy. J Urol.

[10] Connolly JA, Shinohara K, Presti JC, Carroll PR (1996). Local recurrence after radical prostatectomy: characteristics in size, location, and relationship to prostate-specific antigen and surgical margins. Urology.

[11] Poortmans P, Bossi A, Vandeputte K, Bosset M, Miralbell R, Maingon P, Boehmer D, Budiharto T, Symon Z, van den Bergh AC, Scrase C, Van Poppel H, Bolla M, Group ERO (2007). Guidelines for target volume definition in post-operative radiotherapy for prostate cancer, on behalf of the EORTC Radiation Oncology Group. Radiother Oncol.

[12] Sidhom MA, Kneebone AB, Lehman M, Wiltshire KL, Millar JL, Mukherjee RK, Shakespeare TP, Tai KH (2008). Post-prostatectomy radiation therapy: consensus guidelines of the Australian and New Zealand Radiation Oncology Genito-Urinary Group. Radiother Oncol.

[13] Wiltshire KL, Brock KK, Haider MA, Zwahlen D, Kong V, Chan E, Moseley J, Bayley A, Catton C, Chung PW, Gospodarowicz M, Milosevic M, Kneebone A, Warde P, Menard C (2007). Anatomic boundaries of the clinical target volume (prostate bed) after radical prostatectomy. Int J Radiat Oncol Biol Phys.

[14] Michalski JM, Lawton C, El Naqa I, Ritter M, O'Meara E, Seider MJ, Lee WR, Rosenthal SA, Pisansky T, Catton C, Valicenti RK, Zietman AL, Bosch WR, Sandler H, Buyyounouski MK, Menard C (2010). Development of RTOG consensus guidelines for the definition of the clinical target volume for postoperative conformal radiation therapy for prostate cancer. Int J Radiat Oncol Biol Phys.

[15] Croke J, Malone S, Roustan Delatour N, Belanger E, Avruch L, Morash C, Kayser C, Underhill K, Spaans J (2012). Postoperative radiotherapy in prostate cancer: the case of the missing target. Int J Radiat Oncol Biol Phys.

[16] Parker C, Sydes MR, Catton C, Kynaston H, Logue J, Murphy C, Morgan RC, Mellon K, Morash C, Parulekar W, Parmar MK, Payne H, Savage C, Stansfeld J, Clarke NW (2007). Radiotherapy and androgen deprivation in combination after local surgery (RADICALS): a new Medical Research Council/National Cancer Institute of Canada phase III trial of adjuvant treatment after radical prostatectomy. BJU Int.

[17] Marks LB, Yorke ED, Jackson A, Ten Haken RK, Constine LS, Eisbruch A, Bentzen SM, Nam J, Deasy JO (2010). Use of normal tissue complication probability models in the clinic. Int J Radiat Oncol Biol Phys.

[18] Davis BJ, Pisansky TM, Wilson TM, Rothenberg HJ, Pacelli A, Hillman DW, Sargent DJ, Bostwick DG (1999). The radial distance of extraprostatic extension of prostate carcinoma: implications for prostate brachytherapy. Cancer.

[19] Sohayda C, Kupelian PA, Levin HS, Klein EA (2000). Extent of extracapsular extension in localized prostate cancer. Urology.

[20] Wang JK, R. Choi, S. Pettaway, C. Choi, H. Hobbs, B. Occena, M. McGuire, S. Pugh, T. Hoffman, K. Mahmood, U. Kuban, D. Local recurrence map to guide target volume delineation after radical prostatectomy. Practical Radiation Oncology. doi: http://dx.doi.org/10.1016/j.prro.2014.02.007 10.1016/j.prro.2014.02.007 25407875

[21] Chang JH, Lim Joon D, Nguyen BT, Hiew CY, Esler S, Angus D, Chao M, Wada M, Quong G, Khoo V (2014). MRI scans significantly change target coverage decisions in radical radiotherapy for prostate cancer. J Med Imaging Radiat Oncol.

[22] Tanaka H, Hayashi S, Ohtakara K, Hoshi H, Iida T (2011). Usefulness of CT-MRI fusion in radiotherapy planning for localized prostate cancer. J Radiat Res.

[23] Ost P, De Meerleer G, Vercauteren T, De Gersem W, Veldeman L, Vandecasteele K, Fonteyne V, Villeirs G (2011). Delineation of the postprostatectomy prostate bed using computed tomography: interobserver variability following the EORTC delineation guidelines. Int J Radiat Oncol Biol Phys.

[24] Barentsz JO, Richenberg J, Clements R, Choyke P, Verma S, Villeirs G, Rouviere O, Logager V, Futterer JJ, European Society of Urogenital R (2012). ESUR prostate MR guidelines 2012. Eur Radiol.

[25] Mullerad M, Hricak H, Kuroiwa K, Pucar D, Chen HN, Kattan MW, Scardino PT (2005). Comparison of endorectal magnetic resonance imaging, guided prostate biopsy and digital rectal examination in the preoperative anatomical localization of prostate cancer. J Urol.

[26] Khoo VS, Joon DL. New developments in MRI for target volume delineation in radiotherapy. The British journal of radiology. 2006;79 Spec No 1:S2-15. 10.1259/bjr/41321492 16980682

[27] Jackson AS, Parker CC, Norman AR, Padhani AR, Huddart RA, Horwich A, Husband JE, Dearnaley DP (2005). Tumour staging using magnetic resonance imaging in clinically localised prostate cancer: relationship to biochemical outcome after neo-adjuvant androgen deprivation and radical radiotherapy. Clin Oncol.

[28] Khoo EL, Schick K, Plank AW, Poulsen M, Wong WW, Middleton M, Martin JM (2012). Prostate contouring variation: can it be fixed?. Int J Radiat Oncol Biol Phys.

[29] Thompson J, Lawrentschuk N, Frydenberg M, Thompson L, Stricker P, USANZ (2013). The role of magnetic resonance imaging in the diagnosis and management of prostate cancer. BJU Int.

[30] Tan N, Margolis DJ, McClure TD, Thomas A, Finley DS, Reiter RE, Huang J, Raman SS (2012). Radical prostatectomy: value of prostate MRI in surgical planning. Abdom Imaging.

[31] Prostate Cancer: Diagnosis and Treatment. 2014. NICE guidelines [CG175], https://www.nice.org.uk/guidance/cg175. Accessed May 2014 2014.

[32] Eberhardt SC, Carter S, Casalino DD, Merrick G, Frank SJ, Gottschalk AR, Leyendecker JR, Nguyen PL, Oto A, Porter C, Remer EM, Rosenthal SA (2013). ACR Appropriateness Criteria prostate cancer-pretreatment detection, staging, and surveillance. J Am Coll Radiol.

